# Production and Characterization of Potentially Symbiotic Acerola Ice Cream with Partially Hydrolyzed Guar Gum and Added *Lacticaseibacillus rhamnosus* GG

**DOI:** 10.3390/foods15122186

**Published:** 2026-06-17

**Authors:** Mariana Estrela de Andrade, Isabela Soares Magalhães, Maurilio Lopes Martins, Fabiana de Oliveira Martins, Eliane Maurício Furtado Martins, Luana Lucas Dutra, Bruno Ricardo de Castro Leite Júnior

**Affiliations:** 1 Department of Food Science and Technology, Federal Institute of Education, Science and Technology of Southeast Minas Gerais, Rio Pomba Campus, Avenue Doctor José Sebastião da Paixão—Lindo Vale, Rio Pomba 36180-000, MG, Brazil; marianaestreladeandrade@gmail.com (M.E.d.A.); maurilio.martins@ifsudestemg.edu.br (M.L.M.); fabiana.martins@ifsudestemg.edu.br (F.d.O.M.); eliane.martins@ifsudestemg.edu.br (E.M.F.M.); 2 Department of Food Technology, Federal University of Viçosa, Av. Peter Henry Rolfs, s/n, University Campus, Viçosa 36570-900, MG, Brazil; isabela.magalhaes@ufv.br; 3 Department of Agricultural Microbiology, Federal University of Viçosa, Viçosa University Campus, Viçosa 36570-900, MG, Brazil; luanalucasdutra@gmail.com

**Keywords:** frozen dessert, acerola pulp, partially hydrolyzed guar gum, *Lacticaseibacillus rhamnosus* GG, symbiotic

## Abstract

This study aimed to develop low-fat acerola ice creams enriched with partially hydrolyzed guar gum (PHGG) at concentrations of 6% and 12% and supplemented with *Lacticaseibacillus rhamnosus* GG. Three formulations were prepared by partially or totally replacing fat with PHGG. After preparation, the ice creams were stored at −18 °C and evaluated over 180 days. Physicochemical analyses showed no differences in pH, acidity, moisture, or ash content among the samples. However, soluble solids and fat content varied depending on the PHGG level. The melting rate remained stable, while overrun increased proportionally with PHGG incorporation. Rheologically, PHGG addition significantly enhanced consistency. Microbiological analyses confirmed that all samples complied with safety standards. The ice creams exhibited symbiotic potential, maintaining *L. rhamnosus* GG viability > 8 log CFU/g for up to 180 days. In simulated gastrointestinal resistance tests, probiotic survival increased with PHGG concentration. After one day, counts during the enteric phase were 3.87, 6.20, and 6.08 log CFU/g for 0%, 6%, and 12% PHGG, respectively. After 180 days, the counts were 1.98, 4.41, and 3.25 log CFU/g, with corresponding survival rates of 47%, 84%, and 78% after one day, and 36%, 53%, and 42% after 180 days. Sensory analysis with 121 untrained panelists revealed no significant differences in aroma and taste. However, samples with higher fat content were better accepted in terms of appearance, texture, and purchase intent. Overall, partial fat replacement with PHGG proved effective in reducing fat while maintaining quality and enhancing probiotic stability, supporting its potential for functional low-fat foods.

## 1. Introduction

The food industry is constantly seeking innovations that align health benefits with consumer appeal. The development of ice creams with functional attributes has emerged as a market trend, as this product is widely appreciated across different ages and cultures [1]. Traditionally, ice cream is considered a high-fat food [2], and excessive intake of this nutrient has been associated with health conditions such as obesity, cardiovascular diseases, diabetes, and certain types of cancer [3]. In this context, the formulation of low-fat ice creams represents a promising alternative for individuals with obesity or related complications, especially when combined with functional ingredients [4].

Among the innovations aimed at healthier eating, functional foods containing prebiotics and probiotics have gained attention for their positive effects on gut health. Prebiotics are non-digestible compounds that serve as substrates for fermentation by the gut microbiota, offering benefits such as modulation of the microbiome, reduction of inflammatory processes, and improvement of digestive health. Furthermore, they support the growth and activity of probiotics, thereby enhancing their beneficial effects [5].

One notable prebiotic ingredient is partially hydrolyzed guar gum (PHGG) [6]. PHGG is a water-soluble dietary fiber that does not form gels and exhibits relatively low viscosity compared to native guar gum, while maintaining prebiotic functionality [6,7]. In contrast to commonly used prebiotics such as inulin and fructooligosaccharides (FOS), which are highly fermentable and can significantly increase viscosity or promote gel-like structures, PHGG provides a more moderate impact on the rheological properties of food systems [6]. This characteristic is particularly advantageous in ice cream formulations, where excessive viscosity may hinder air incorporation and negatively affect overrun and texture [8]. Furthermore, PHGG has been widely studied for its physiological and clinical benefits; however, its application in frozen dairy systems remains limited compared to other prebiotics. Therefore, investigating the use of PHGG in ice cream formulations represents an opportunity to explore its functional and technological potential in this type of product.

Probiotics, on the other hand, are live microorganisms that, when administered in adequate amounts, confer health benefits to the host [9]. These organisms can colonize the gastrointestinal tract and exert beneficial effects by inhibiting or suppressing pathogenic microbes [10,11]. *Lacticaseibacillus rhamnosus* GG is among the most extensively studied probiotic strains [12], known for its high adhesion capacity in vitro, strong antimicrobial activity, and notable resistance to gastric acidity [13]. Currently, symbiotic foods—those combining prebiotics and probiotics—are gaining popularity, driving consumer interest and supermarket demand [14].

In the ice cream sector, traditional flavors such as chocolate, strawberry, vanilla, coconut, and pineapple are already well established, but there remains a gap regarding acerola-flavored products. Acerola (*Malpighia emarginata*) is appreciated for its aroma and flavor and stands out as one of the richest natural sources of ascorbic acid, reaching up to 1678 mg/100 g of pulp [15]. In addition to its high vitamin C content, acerola presents a diverse nutritional composition, including carbohydrates (approximately 7.69 g/100 g), proteins (0.4 g/100 g), lipids (0.3 g/100 g), dietary fiber (1.1 g/100 g), and ash (0.2 g/100 g). The fruit is also rich in organic acids, with malic acid being one of the predominant compounds (0.46–1.10 g/100 g), accounting for more than 30% of the total organic acid content [16]. Moreover, acerola contains several bioactive compounds, such as phenolic compounds and carotenoids, which contribute to its antioxidant properties. It is important to note that the composition of acerola may vary depending on factors such as cultivar, maturation stage, environmental conditions, and cultivation practices [17]. Therefore, this study aimed to develop a low-fat, potentially symbiotic acerola ice cream enriched with PHGG and *L. rhamnosus* GG. The formulation strategy was based on the direct replacement of fat by PHGG, aiming to simultaneously reduce lipid content and incorporate a prebiotic component into the system. This approach not only enhances the nutritional profile of the product but also promotes symbiotic potential by combining the health benefits of dietary fiber and probiotic microorganisms. Additionally, this formulation represents an innovative and health-oriented alternative in the frozen dessert segment, aligning with the growing consumer demand for functional foods.

## 2. Materials and Methods

### 2.1. Production of Acerola Ice Cream with Partial and Total Fat Replacement by PHGG and Addition of Lacticaseibacillus rhamnosus GG

Acerola ice cream was produced by partially and totally replacing fat with Partially Hydrolyzed Guar Gum (PHGG), resulting in three different formulations. The following ingredients were used: industrial acerola pulp (Polpas Roma, Guidoval, MG, Brazil), skim milk powder (Leitino^®^, Contagem, MG, Brazil), pasteurized cream (58% fat) (Laticínios Boreal, Rio Pomba, MG, Brazil), granulated sugar, stabilizer and emulsifier for ice cream (Progel S200 Premium, Proregi, Rio Pomba, MG, Brazil), PHGG (Sunfiber^®^, TAIYO International, Inc., Edina, MN, USA), a partially hydrolyzed guar gum composed of galactomannan polysaccharides derived from the endosperm of *Cyamopsis tetragonolobus* seeds, with a molecular weight range of approximately 1–100 kDa and characterized by high water solubility and low viscosity, and *Lacticaseibacillus rhamnosus* GG (Manipular Compounding Pharmacy, Rio Pomba, MG, Brazil).

Each formulation was composed of specific combinations and quantities of the aforementioned ingredients (Table 1), which were mixed in different proportions to prepare the ice cream mixes—a homogeneous liquid blend of all components. These mixes were standardized according to fat and PHGG content, as detailed in Table 1.

The ice cream mixes were pasteurized at 70 °C for 30 min using a Thermomix^®^ (Vorwerk, Thermomix TM31, Wuppertal, Germany). The temperature was then reduced to 20 °C using an ice bath with manual stirring to allow inoculation with *Lacticaseibacillus rhamnosus* GG at an initial concentration of 11 log CFU/g, aiming to achieve a final count between 8–9 log CFU/g in the product.

The mixes were then matured under refrigeration at 4 °C for 24 h. Following maturation, churning and freezing were carried out using an ice cream maker (V5, FortFrio, Betim, MG, Brazil). The ice creams were then packaged in polyethylene containers and stored at −18 °C.

### 2.2. Evaluation of the Impact of Partial and Total Fat Replacement by PHGG on the Physicochemical and Technological Characteristics of the Ice Cream

Before analysis, the ice cream samples were thawed under refrigeration (4 °C) and homogenized to ensure uniformity for physicochemical analyses. For technological properties (melting rate, overrun, and apparent density), the analyses were performed directly on the frozen samples without prior thawing. One day after production, triplicate analyses were performed to evaluate pH, titratable acidity, total solids, moisture, protein, lipid, ash, and carbohydrate contents. Additionally, melting rate, overrun, and apparent density were determined both one day after production and after 180 days of storage.

#### 2.2.1. Determination of pH, Titratable Acidity, and Total Soluble Solids

According to AOAC [18] methods, pH was measured using a potentiometer (Tecnopon NT PHM, Piracicaba, SP, Brazil). Titratable acidity was expressed as grams of lactic acid per 100 g of ice cream. Total soluble solids were determined using a digital refractometer (Milwaukee, MA871, Cluj-Napoca, Romania).

#### 2.2.2. Determination of Moisture, Protein, Lipid, Ash, and Carbohydrate Contents

Moisture and ash contents were determined by gravimetric methods, and protein content was assessed using the Kjeldahl method, applying a nitrogen-to-protein conversion factor of 6.38, as described in AOAC [18]. Lipid content was determined according to the Bligh and Dyer method, which involves cold extraction using a chloroform, methanol, and water mixture, allowing efficient separation of total lipids, as described by Cecchi [19]. Carbohydrate content was calculated by difference, subtracting the sum of moisture, protein, lipid, and ash percentages from the total.

#### 2.2.3. Melting Rate, Overrun, and Apparent Density

**Figure 1:**
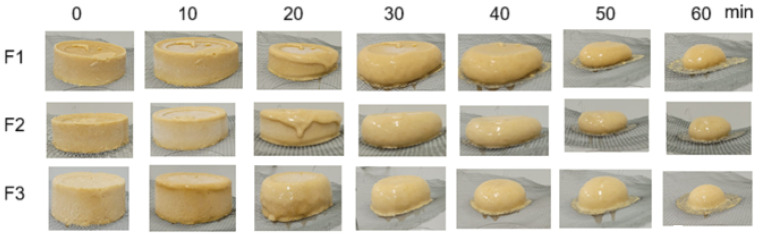
Melting profile of ice creams after 1 day of manufacture. Comparison of melting times evaluated every 10 min. Columns represent different time points, while lines compare formulations F1, F2, and F3. PHGG: Partially hydrolyzed guar gum. F1: 0% PHGG; F2: 6% PHGG; F3: 12% PHGG.

Melting rate was determined using a method adapted from Granger et al. [20]. Briefly, ice cream samples were placed on a mesh screen at room temperature, and the melted fraction was collected over time. The time to first drop was recorded as the time (min) elapsed from the beginning of the test until the first drop of melted ice cream was observed and collected. In addition, photographs of the samples were taken at 10 min intervals (0, 10, 20, 30, 40, 50, and 60 min) throughout the melting test to visually evaluate and compare the melting profiles of the formulations (Figure 1). Overrun was measured one day after ice cream production based on the difference between the mix and final product weights, according to Equation (1) [21]. Apparent density was calculated as the ratio between the mass and volume of the ice cream, expressed in g/L.

(1) $$\%\;Overrun=\frac{Mix\;weight-Ice\;cream\;weight}{Ice\;cream\;weight}\times 100$$

### 2.3. Rheological Properties of Ice Cream Mixes

Rheological analyses were performed using a concentric cylinder rheometer (R/S Plus SST 2000, Brookfield Engineering Laboratories, Middleboro, MA, USA) with an interface connected to a microcomputer equipped with RHEO Software CALC V1.1, following the procedures described by Paula et al. [22]. Flow curves were obtained using a CC45 sensor with a shear rate ranging from 0 to 300 s^−1^, applied in four consecutive ramps (upward, downward, upward, and downward), each lasting 3 min with measurements taken every 4 s to eliminate thixotropic effects. During the experiments, the ice cream mixes samples were maintained at 7 ± 0.1 °C. The data were fitted to the Ostwald–de Waele model (Power Law, Equation (2)) using Curve Expert Professional Software version 2.2.0. The consistency index (K) and flow behavior index (*n*) were used to calculate the apparent viscosity (η_app_, Pa·s) at shear rates of 10, 50, and 100 s^−1^.

(2) $$\sigma ={K\cdot \gamma }^{n}\;$$

where σ is the shear stress (Pa), K is the consistency index (Pa·s*^n^*), γ is the shear rate (s^−1^), and n is the flow behavior index (dimensionless).

### 2.4. Evaluation of the Impact of Partial and Total Fat Replacement by PHGG on the Viability and Gastrointestinal Resistance of L. rhamnosus GG Under In Vitro Simulated Conditions

#### 2.4.1. Probiotic Viability

The viability of *Lacticaseibacillus rhamnosus* GG in the ice cream formulations was assessed through standard plate counting using Man, Rogosa and Sharpe (MRS) agar (Merck, Darmstadt, Germany), according to the method described by Richer and Vedamuthu [23]. Samples were evaluated after 1, 45, 90, 135, and 180 days of storage at −18 °C. Following deep inoculation, the plates were incubated at 37 °C for 72 h under microaerophilic conditions, using an anaerobic jar. Colony counts were then performed, and the results were expressed as Colony Forming Units per gram (CFU/g).

#### 2.4.2. In Vitro Simulation of Gastrointestinal Conditions

The in vitro simulation of gastrointestinal conditions comprised three stages, gastric, enteric I, and enteric II, and was conducted following the methodology described by Bedani et al. [24]. Analyses were performed both immediately after ice cream preparation and after 180 days of storage at −18 °C.

Ice cream samples from each formulation were diluted in 0.85% saline solution at a 1:10 ratio (10^−1^ dilution), and 10 mL aliquots were transferred into 100 mL dilution bottles. The pH was adjusted to 2.3–2.6 using 1 mol/L HCl (Impex, Diadema, São Paulo, Brazil), followed by the addition of 3 g/L of pepsin (isolated from porcine gastric mucosa, Sigma-Aldrich, St. Louis, MO, USA) and 0.9 mg/L of lipase (Amano lipase G, isolated from *Penicillium camemberti*, Sigma-Aldrich). Samples were then incubated at 37 °C for 2 h at 150 rpm in a shaking incubator (Tecnal TE-424, Piracicaba, São Paulo, Brazil).

After gastric phase incubation, the small intestine simulation (enteric phase I) was initiated by adjusting the pH to 5.4–5.7 using sodium phosphate solution at pH 12 [prepared with 150 mL of 1 mol/L NaOH and 14 g of NaH_2_PO_4_·2H_2_O (Vetec, Duque de Caxias, RJ, Brazil)]. This solution also contained bile (10.0 g/L, bovine bile, Sigma-Aldrich) and pancreatin (1.0 g/L, from porcine pancreas, Sigma-Aldrich). Incubation continued under the same conditions for an additional 2 h.

Following 4 h of total incubation, the large intestine simulation (enteric phase II) was performed by adjusting the pH to 6.8–7.2 using the same alkaline solution containing bile and pancreatin. A third incubation phase under the same conditions was conducted, totaling 6 h of digestion simulation. At the end of each incubation cycle (2, 4, and 6 h), corresponding to the gastric and enteric phases I and II, probiotic viability was assessed via standard plate counts using MRS agar by deep plating. Plates were incubated at 37 °C for 72 h in anaerobic jars. The results were expressed in log CFU/g. In addition, the probiotic survival rate (%) was calculated as the ratio between the logarithmic values of CFU at the end (N_1_) and at the beginning (N_0_) of the in vitro assay (Equation (3)).

(3) $$Survival\;rate\;(\%)=\frac{log\;CFU_{n0}}{logCFU_{n0}}\times 100$$

where CFUn^1^ is the probiotic cell count at the end of the in vitro assay and CFUn^0^ is the cell count before the assay.

### 2.5. Microbiological Analysis of Ice Cream

Microbiological analyses were performed after 5 days of storage at −18 °C to verify compliance with the applicable microbiological legislation and confirm the microbiological safety of the formulations prior to subsequent analyses. The presence of Enterobacteriaceae [25], coagulase-positive staphylococci [26], and *Salmonella spp*. [27] was assessed in samples obtained from three independent production batches. Results were compared against the microbiological standards established by Brazilian Normative Instruction N°. 161 [28].

### 2.6. Sensory Evaluation and Purchase Intent of Ice Creams

This research was approved by the Human Research Ethics Committee (CAAE: 78860724.6.0000.5588). Participants were volunteers who signed an informed consent form. The acceptance test included 121 untrained panelists aged over 18 years. Participants were informed about the formulation ingredients and instructed not to participate if they had any allergies, intolerances, or dietary restrictions related to the product.

Ice creams stored for 30 days were served to participants, who received a questionnaire to complete. The samples were coded with three-digit numbers and served randomly in plastic cups (approximately 25 g per portion). A 200 mL cup of water was provided for oral rinsing between samples.

The questionnaire included demographic and ice cream consumption habit questions, as well as a 9-point hedonic scale ranging from “like extremely” (score 9) to “dislike extremely” (score 1) to evaluate color, flavor, texture, aroma, and overall impression. Purchase intent was also assessed using a 5-point scale from “definitely would buy” (score 5) to “definitely would not buy” (score 1). The sensory evaluation followed the procedures described by Minim [29].

### 2.7. Statistical Analysis

Ice cream processing was performed in three replicates, and all analyses were conducted in triplicate. Results were expressed as means ± standard deviations. The statistical design used was a randomized block design (RBD). Data were first tested for normality, followed by analysis of variance (ANOVA). Tukey’s test (*p* < 0.05) was applied to compare means using the Statistical Analysis System software (SAS Institute, Cary, NC, USA; version 9.2).

## 3. Results and Discussion

### 3.1. Evaluation of the Impact of Partial and Total Fat Replacement by PHGG on the Physicochemical Characteristics and Proximate Composition of the Formulated Products

The ice cream formulations showed no significant differences (*p* > 0.05) in titratable acidity or pH with fat replacement, nor as a function of storage time (Table 2). This result was expected, given that all three formulations contained identical proportions of acerola, which is the main component directly influencing the acidity of the product. Furthermore, the acerola pulp used in the present study exhibited a pH of 3.5, which is consistent with the values reported by Viana et al. [30] for this fruit. Similar pH values were also reported by Favaro-Trindade et al. [31] in fermented acerola ice creams, reinforcing the influence of acerola pulp on the acidity characteristics of frozen dairy desserts. Despite the high acidity and low pH, the ice creams showed no signs of protein instability, even after the pasteurization process. This outcome highlights the effectiveness of the stabilizer Progel S200 Premium in maintaining the protein matrix stability of the product.

On the other hand, total soluble solids (TSS) increased (*p* < 0.05) with the addition of PHGG, with no influence from storage time. The sample with the highest fat content (F1) showed the lowest TSS values (<27.1 °Brix), whereas the fat-free sample (F3) exhibited the highest TSS values (>34 °Brix), due to the total fat replacement by PHGG (Table 2). This result was expected, since PHGG is a water-soluble carbohydrate that contributes to increased soluble solids, whereas fat, being water-insoluble, has no impact on this parameter.

Moisture (62.5% to 64%) and ash contents (0.90% to 1.01%) did not differ significantly among the formulations or during storage (*p* > 0.05), indicating that the formulation balance to maintain total solids content was effective. Furthermore, the partial or total replacement of fat by PHGG did not affect ash content (Table 3).

Protein content also did not vary significantly among the formulations (*p* > 0.05), with an average value of approximately 4%, due to the standardized amount of skimmed milk powder used. However, a slight reduction (<1%) was observed during storage (*p* < 0.05) (Table 3). This decrease is unlikely to be associated with actual protein degradation, since proteolytic and microbial activities are minimal under frozen conditions. Instead, it may be related to physicochemical and structural changes occurring during frozen storage. Ice recrystallization and temperature fluctuations can promote modifications in the frozen matrix, including phase separation and destabilization of the air cell structure, which are known to affect the stability and distribution of components in frozen desserts [21,32,33]. In addition, structural destabilization phenomena such as shrinkage and collapse of the frozen foam matrix have been reported during storage, being strongly influenced by protein functionality at interfaces [34,35]. Furthermore, interactions between milk proteins and PHGG may influence protein distribution within the frozen matrix. Similar protein–polysaccharide interactions have been reported for guar gum and milk protein systems, affecting network formation, microstructure, and physicochemical stability [36,37,38]. These effects may alter protein extractability during analysis, leading to slight variations in the measured protein content.

In contrast, total carbohydrate content increased (*p* < 0.05) with decreasing fat content, with no effect from storage time (*p* > 0.05). Conversely, lipid content decreased (*p* < 0.05) proportionally to the fat replacement by PHGG, with differences among formulations but without expressive variation during storage (Table 3). The increase in total carbohydrate content was expected, since fat reduction was compensated by the addition of PHGG, which is classified as a dietary fiber. As total carbohydrates were calculated by difference, this fraction includes all non-lipid components, including dietary fiber. Therefore, the incorporation of PHGG directly contributed to the increase in total carbohydrate content as fat levels decreased.

Although the total fiber content in the ice creams was not determined, it is expected that fat replacement by PHGG resulted in a product with high fiber content, directly impacting its nutritional profile. This effect is considered positive, as fat is one of the main sources of dietary calories, justifying strategies for its reduction in foods [39], especially for individuals requiring fat-restricted diets. Moreover, this modification becomes even more relevant in the context of excessive intake of salt, fat, and sugar among children and adolescents [40], encouraging healthier choices.

### 3.2. Melting Rate, Overrun, and Density of the Ice Creams

The addition of PHGG significantly increased (*p* < 0.05) the overrun of the ice creams (Table 4). The formulation without PHGG showed an overrun of 27.8%, while the formulation with reduced fat and PHGG (F2) reached 42%. In the formulation in which fat was fully replaced by PHGG (F3), the overrun reached 89%, a value comparable to that reported by Kowalczyk et al. [41], who used inulin and apple fiber in sheep milk-based ice creams. Similarly, Pimisa et al. [42] observed higher overrun values in ice creams supplemented with pectin extracted from passion fruit peel. This increase is commonly associated with changes in the rheological properties of the mix, particularly increased viscosity, which may favor air incorporation and retention during the freezing process [43]. However, overrun in ice cream is a multifactorial property influenced not only by viscosity, but also by interfacial phenomena and fat destabilization. In conventional systems, partial coalescence of fat globules plays a key role in stabilizing air cells. In the present study, the high overrun observed in the fat-free formulation (F3) suggests that mechanisms other than fat destabilization contributed to air incorporation and stability.

In this context, the presence of PHGG may have enhanced the viscosity of the serum phase and increased water binding capacity, which can reduce drainage and improve air retention during freezing. Additionally, modification of the continuous phase may contribute to air cell stabilization, thereby improving the overall stability of the aerated structure [44]. Therefore, the observed increase in overrun is likely the result of combined effects involving rheological properties and matrix stabilization, rather than viscosity alone.

Despite the differences observed in overrun, the melting rate did not vary significantly (*p* > 0.05) among treatments and storage times (Figure 1 and Table 4). On the first day, the formulations presented melting rates ranging from 1.82 to 2.06 g/min. The time for the first drop formation ranged from 8.2 to 10.0 min, with no significant difference among formulations (*p* > 0.05) (Table 4). These results indicate that, under the conditions evaluated, fat replacement by PHGG did not markedly influence melting behavior, likely due to the balance of multiple structural and physicochemical factors. This behavior suggests that, although differences in composition and overrun were observed, multiple factors may have contributed in a compensatory manner. While higher overrun is generally associated with faster melting due to increased air incorporation, the presence of PHGG likely enhanced water retention and increased the viscosity of the continuous phase, which can slow down melting [44,45]. In addition, structural factors such as fat destabilization, air cell distribution, and matrix organization also play important roles in melting resistance [44]. Therefore, the balance between these competing effects may explain the similar melting rates observed among the formulations.

The density of the ice creams was influenced by the replacement of fat with PHGG. A progressive decrease in density was observed as fat content decreased. Sample F1 (high fat content) showed the highest mean density (919 g/L), followed by F2 (6% fat) with 811 g/L, and F3 (free-fat) with 631 g/L. These results are consistent with the observed increase in overrun, since greater air incorporation typically results in lower density. Thus, the lower density observed in F3 reflects a higher level of air incorporation and a less compact structure compared to the other formulations. All samples met the requirements of RDC Resolution No. 266/2005 [46], which establishes a minimum density of 475 g/L for ice cream.

Fat is known to play an important role in the texture and melting properties of ice cream [47]. Nevertheless, in the present study, no significant differences in melting rate were observed among formulations with varying PHGG levels. Liu et al. [48] reported that fat destabilization may have a greater impact on melting behavior than fat content itself. In this context, the absence of differences in melting rate suggests that the replacement of fat by PHGG did not substantially alter the factors governing melting resistance under the evaluated conditions.

Although the results indicate that PHGG influenced overrun and density, its effect on melting behavior was not significant. Similar observations have been reported for other prebiotic ingredients, although their impact appears to be dependent on their physicochemical characteristics and interactions within the matrix [49]. Nevertheless, the results obtained from overrun, density, and melting behavior provide consistent and practically relevant insights into the performance of the ice cream formulations, particularly regarding air incorporation and product stability.

### 3.3. Rheological Properties Evaluation

The experimental data showed an excellent fit to the Ostwald–de Waele model, with coefficients of determination (R^2^ > 0.99), indicating high reliability in describing the rheological behavior of the samples.

**Figure 2:**
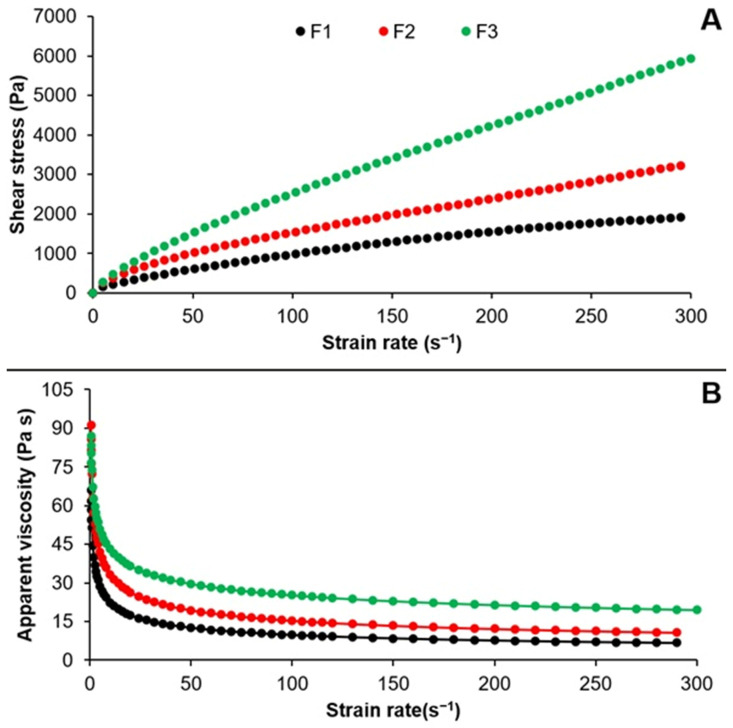
Shear stress versus shear rate and apparent viscosity as a function of shear rate. (**A**) Shear stress (σ, Pa) as a function of shear rate (γ, s^−1^) and (**B**) apparent viscosity (η, Pa·s) as a function of shear rate (γ, s^−1^) at 7 °C for F1: 0% PHGG; F2: 6% PHGG; and F3: 12% PHGG.

The rheological modeling, based on the consistency index (k), flow behavior index (*n*), and apparent viscosity at different shear rates (γ = 10 s^−1^, 50 s^−1^, and 100 s^−1^), allowed for the assessment of the effect of fat replacement by PHGG on the flow characteristics of the product. The results highlighted the impact of fat substitution with PHGG on the product’s rheology, revealing differences between the formulations (Table 5). Furthermore, Figure 2 presents the complete flow curves (Figure 2A) and apparent viscosity profiles (Figure 2B) over the entire shear rate range evaluated, illustrating the shear-thinning behavior of the formulations. In contrast, Table 5 summarizes the rheological parameters obtained from the Ostwald–de Waele model and the apparent viscosity values at selected shear rates, allowing a quantitative comparison among samples.

Significant differences (*p* < 0.05) were observed in the consistency index (k), with formulation 1 (0% PHGG) showing a lower value (51.3 Pa·s*^n^*) compared to formulations 2 and 3 (6% and 12% PHGG), which exhibited higher values (72.2 and 73.8 Pa·s*^n^*, respectively). These results indicate that the partial or total replacement of fat with PHGG increased the viscosity of the ice cream mix, resulting in a more consistent texture. The flow behavior index (*n*) was significantly higher in the fat-free ice cream mix with 12% PHGG (0.77) compared to the mixes containing 6% and 0% PHGG (0.64 and 0.66, respectively). Despite these variations, all formulations exhibited pseudoplastic behavior (*n* < 1).

Accordingly, apparent viscosity decreased as shear rate increased in all samples, indicating progressive alignment and reduced resistance to flow under shear conditions. Significant differences (*p* < 0.05) were found among the samples. The fat-free formulation with PHGG (F3) showed higher apparent viscosity values: up to 92% at 10 s^−1^, 135% at 50 s^−1^, and 157% at 100 s^−1^ when compared to the high-fat formulation without PHGG (F1), indicating that fat removal and replacement with PHGG increased flow resistance. Although PHGG has a relatively low viscosity, its thickening effect on the ice cream mixes was evident.

The increased viscosity of the ice cream mix can be explained by intermolecular interactions between PHGG and matrix components, contributing to the formation of a more structured hydrodynamic network. According to Qi et al. [50] PHGG can act as a glycosyl donor in protein glycation reactions and may interact with lipid droplets, contributing to a more organized network structure. Furthermore, its hydrophilic polysaccharide chains have a high affinity for water [7], promoting increased water retention and reduced molecular mobility in the system. This effect increases flow resistance and influences the consistency index (k) and flow behavior index (*n*), particularly at low shear rates.

These rheological modifications are also relevant to the physical performance of the ice cream system. Increased viscosity of the continuous phase may reduce air bubble coalescence and drainage, thereby improving air incorporation and retention during freezing. In this context, the higher values of consistency index (k) observed in PHGG-containing formulations are consistent with the increased overrun and reduced density reported in Section 3.2. Thus, the rheological behavior of the mixes plays a key role in determining the structural stability and aeration capacity of the final product.

Previous studies have shown that PHGG can modify the structure of food matrices, as reported by Mudgil et al. [51]. In the present study, PHGG contributed to increased consistency, a result similar to that reported by Akalin et al. [52] in ice creams enriched with apple and orange fibers. The increase in viscosity observed in the PHGG-containing formulations may be attributed to the rheological properties of this fiber, which, despite having lower viscosity in aqueous media compared to traditional guar gum, still significantly contributed to the thickening of the system relative to the control formulation without the additive.

### 3.4. Impact of Partial and Total Fat Replacement by PHGG on the Viability and In Vitro Simulated Gastrointestinal Resistance of L. rhamnosus GG in Ice Creams

#### 3.4.1. Probiotic Viability

**Figure 3:**
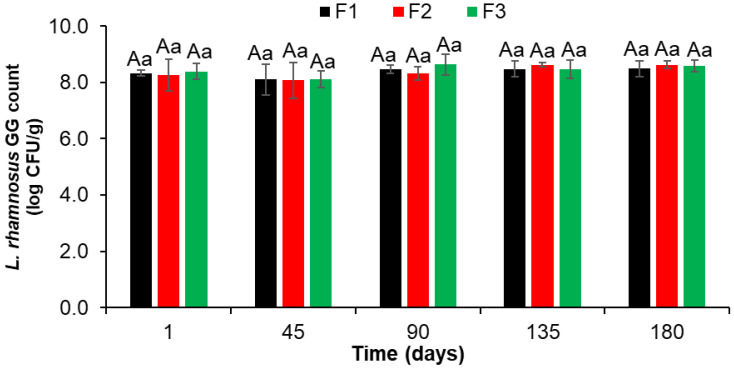
Viability of *Lacticaseibacillus rhamnosus* GG in acerola ice cream with partial and total fat replacement by PHGG over 180 days of storage at −18 °C. Different uppercase letters indicate significant differences between samples at the same time point according to Tukey’s test at a 5% significance level (*p* < 0.05). Lowercase letters indicate significant differences over time for the same sample based on the same statistical test. PHGG: Partially hydrolyzed guar gum. F1: 0% PHGG; F2: 6% PHGG; F3: 12% PHGG.

The viability of the probiotic was evaluated at 45-day intervals, and no significant differences (*p* < 0.05) were observed over the 180 days of storage at −18 °C. Throughout the entire period, the average viability values remained above 8 log CFU/g, indicating probiotic stability under the evaluated conditions and throughout the product’s shelf life. Moreover, no significant differences (*p* < 0.05) were observed among the formulations, indicating that the addition of PHGG did not affect the viability of the probiotic (Figure 3). Despite the naturally acidic composition of acerola, the ice cream formulations presented pH values ranging from 4.65 to 4.71, which did not negatively affect probiotic viability. The high survival of *L. rhamnosus* GG throughout storage suggests that the dairy matrix and frozen conditions provided sufficient protection within a matrix rich in organic acids and bioactive compounds.

When added to food products, probiotics must be present in a minimum effective number of viable cells at the time of consumption to exert their health benefits. According to Normative Instruction N°. 284 [53], the minimum required amount of viable probiotic microorganisms in food is 2 × 10^9^ CFU (9.3 log CFU) per day, which should be provided by the daily portion of the product. In this context, the analyzed ice cream samples presented counts exceeding 10 log CFU per 100 g serving, thus meeting regulatory requirements.

Ranadheera et al. [54] consider a count of 6 to 7 log CFU/g as the minimum level necessary to achieve therapeutic effects. The World Gastroenterology Organisation recommends a daily intake of 10^10^ CFU of *Lacticaseibacillus rhamnosus* GG, administered twice a day for both outpatients and hospitalized patients [55]. The viable counts of *L. rhamnosus* GG remained stable across all ice cream formulations over 180 days of frozen storage, demonstrating its resistance to freezing and preservation of probiotic viability. This finding indicates that this strain can be successfully incorporated into frozen food products without compromising its effectiveness. A similar result was reported by Hernández-Riveros et al. [56], who observed an average count of 8.48 log CFU/g after 14 days in ice cream supplemented with *L. rhamnosus* GG and Aguamiel syrup, reinforcing the robustness of this probiotic strain across different food matrices.

#### 3.4.2. Resistance of *Lacticaseibacillus rhamnosus* GG After In Vitro Simulation of Gastrointestinal Conditions When Delivered Through Ice Cream Samples

**Figure 4:**
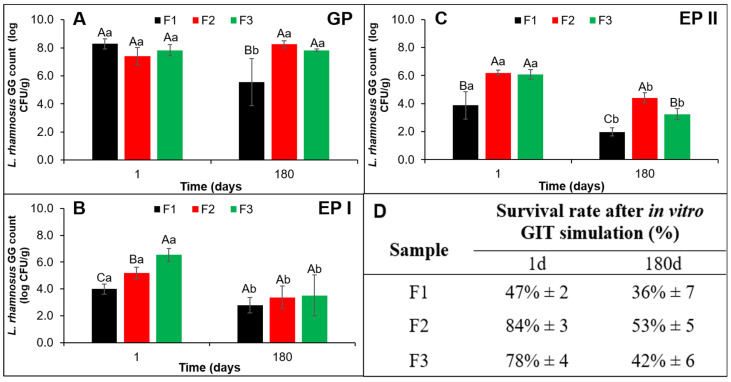
In vitro gastrointestinal resistance (**A**–**C**) and survival rate (**D**) of *Lacticaseibacillus rhamnosus* GG in acerola ice cream with partial and total fat replacement by PHGG over 180 days of storage at −18 °C. Different uppercase letters indicate significant differences between samples at the same time point according to Tukey’s test at a 5% significance level (*p* < 0.05), while different lowercase letters indicate significant differences between time points for the same sample based on the same statistical test. GP: Gastric Phase; EP I: Enteric Phase I; EP II: Enteric Phase II. PHGG: Partially hydrolyzed guar gum. F1: 0% PHGG; F2: 6% PHGG; F3: 12% PHGG.

The results obtained from gastrointestinal simulation should be interpreted independently from storage viability, as they represent different physiological challenges. While frozen storage evaluates probiotic stability within the food matrix, in vitro digestion assays assess the ability of the cells to withstand gastrointestinal stress conditions. In the gastric phase (pH 2.0–2.5), at day 1, the formulations with 0%, 6%, and 12% PHGG showed viable counts of 8.29, 7.40, and 7.83 log CFU/g, respectively, with no significant differences among them (*p* > 0.05). However, after 180 days of storage, the formulation without PHGG (high fat-F1) exhibited a significant reduction (*p* < 0.05), reaching 5.54 log CFU/g. In contrast, the formulations containing 6% and 12% PHGG maintained higher viable counts (8.25 and 7.83 log CFU/g, respectively) (Figure 4). These results suggest that PHGG contributed to increased resistance of *L. rhamnosus* GG under gastric stress conditions, particularly after prolonged storage.

In the enteric phase I (pH 5.4–5.7), significant differences were observed among formulations and throughout storage (*p* < 0.05). After one day, the formulations with 0%, 6%, and 12% PHGG presented viable counts of 3.99, 5.18, and 6.54 log CFU/g, respectively (*p* < 0.05), indicating that PHGG positively influenced probiotic survival at this stage. The addition of prebiotics to the food matrix can enhance the resistance of probiotic microorganisms during gastrointestinal digestion through both physical and metabolic mechanisms. From a structural perspective, non-digestible carbohydrates such as PHGG may contribute to the formation of a protective microenvironment around probiotic cells within the ice cream matrix. This effect is associated with increased water retention, modulation of viscosity, and reduced diffusion of harmful compounds, such as gastric acids and digestive enzymes, thereby limiting cell exposure to stress conditions during digestion [57]. From a metabolic perspective, although PHGG is not significantly metabolized during frozen storage, it may serve as a potential fermentable substrate during gastrointestinal transit, supporting probiotic activity and contributing to improved survival under enteric conditions [57]. These combined effects help explain the higher survival rates observed in formulations containing PHGG, particularly at the beginning of storage. However, after 180 days, *L. rhamnosus* GG counts significantly decreased (*p* < 0.05) in all formulations, with no significant differences among them (*p* > 0.05), ranging from 2.80 to 3.52 log CFU/g (Figure 4). This indicates that the protective effect of PHGG is more pronounced at earlier stages, while long-term storage negatively affects probiotic resistance regardless of formulation. These findings suggest that the improved survival of *L. rhamnosus* GG is associated not only with the compositional characteristics of the formulations but also with matrix-related effects. The presence of PHGG may have contributed to the formation of a more structured and hydrated microenvironment within the ice cream matrix, increasing water retention and creating diffusion barriers that reduce the direct exposure of probiotic cells to acidic and enzymatic stress during gastrointestinal simulation. In addition, possible bacterial adaptive responses to environmental stress may have contributed to the enhanced survival observed in PHGG-containing formulations. Although the spatial distribution of probiotic cells was not directly assessed, the results indicate that matrix-related properties played an important role in enhancing probiotic survival. From a practical perspective, the high viability and improved resistance observed in PHGG-containing formulations represent the most relevant outcome, demonstrating the effectiveness of this ingredient in promoting probiotic stability in frozen systems.

In the enteric phase II (pH 6.8–7.2), significant differences among formulations were also observed after one day of storage (*p* < 0.05), with viable counts of 3.87, 6.20, and 6.08 log CFU/g for the 0%, 6%, and 12% PHGG samples, respectively (Figure 4). These results confirm that PHGG contributed to improved probiotic survival, allowing formulations F2 and F3 to maintain counts above 6 log CFU/g at the end of the simulated digestion process. However, after 180 days, *L. rhamnosus* GG counts decreased significantly (*p* < 0.05) in all samples, reaching 1.98, 4.41, and 3.25 log CFU/g for the 0%, 6%, and 12% PHGG formulations, respectively (Figure 4). These findings reinforce that, although PHGG enhances probiotic resistance under gastrointestinal conditions, its protective effect diminishes over extended storage, indicating the need for additional strategies to maintain probiotic stability over time.

In the present study, it is estimated that the consumption of approximately 100 g of the ice cream formulations would provide, after simulated gastrointestinal transit, *L. rhamnosus* GG counts of 5.87, 8.20, and 8.08 log CFU for the formulations containing 0%, 6%, and 12% PHGG, respectively, at the beginning of storage. After 180 days, these values decreased to 4.0, 6.4, and 5.25 log CFU, respectively.

The data obtained from the gastrointestinal resistance assay demonstrated the viability of *L. rhamnosus* GG in the different ice cream formulations over the storage period. Considering that a minimum concentration of 6.0 log CFU/g at the time of consumption is necessary for a probiotic food to exert its health benefits [58], especially for a standard serving of 100 g, it was observed that the formulations containing 6% and 12% PHGG met this criterion at time zero. The formulation with 6% PHGG maintained counts above 6.0 log CFU even after 180 days of storage, in both enteric phase I and II of the simulated gastrointestinal conditions, standing out as the most effective in protecting and ensuring the viability of the probiotic over time.

The survival rate of *L. rhamnosus* GG was assessed to estimate its relative resistance after processing and exposure to the simulated gastrointestinal tract (Figure 4D). After 1 day of storage, the formulations F1, F2, and F3 (containing 0%, 6%, and 12% PHGG, respectively) showed survival rates of 47%, 84%, and 78%. After 180 days, these values were 36%, 53%, and 42%, respectively. These results further support that PHGG enhances probiotic survival, likely through combined structural and metabolic mechanisms, particularly at earlier stages of storage.

### 3.5. Microbiological Quality of the Developed Ice Creams

All formulations presented microbiological counts within the limits established by Normative Instruction N°. 161 [28], with values below 10^2^ CFU/g for Enterobacteriaceae and coagulase-positive staphylococci, as well as absence of *Salmonella spp*. in 25 g of the product (Table S1, Supplementary Materials). These results confirm the microbiological safety of the products, ensuring their compliance with current legislation and suitability for human consumption.

### 3.6. Sensory Evaluation and Purchase Intention of the Developed Ice Creams

It was found that 81% of the evaluators were young adults between 18 and 25 years old, 67% were undergraduate students, and 53% were women. Moreover, 100% of the panelists reported liking ice cream, and 36% stated they consumed the product at least once a month. For the appearance attribute, a significant difference was observed among the samples (*p* < 0.05). The formulations without PHGG (high-fat-F1) and with 6% fat plus PHGG (F2) received higher scores, being rated between “moderately liked” and “very much liked” (Table 6). In contrast, the formulation with 12% PHGG and no added fat (F3) was rated between “slightly liked” and “moderately liked”.

On the other hand, there were no significant differences among the formulations for aroma and flavor (*p* > 0.05). Aroma was rated between “slightly liked” and “moderately liked” for all samples. Although flavor acceptance did not differ significantly, the F1 sample (high fat, no PHGG) received an average rating between “slightly liked” and “moderately liked,” while the PHGG-containing formulations (F2 and F3) were rated between “neither liked nor disliked” and “slightly liked”.

Regarding texture, a significant difference was observed among the samples (*p* < 0.05). The high-fat formulation without PHGG (F1) achieved the highest score, between “moderately liked” and “very much liked.” The formulation with 6% fat and PHGG (F2) was rated between “slightly liked” and “moderately liked,” whereas the fat-free formulation with 12% PHGG (F3) was rated between “neither liked nor disliked” and “slightly liked.” These results may be associated with the increased overrun observed in the formulations, which likely affected both texture and appearance, possibly influenced by the presence of PHGG. For the overall impression, a significant difference was also observed among the formulations (*p* < 0.05). The high-fat formulation (F1) received the highest score (6.53), significantly differing from the 6% fat formulation (F2), which scored 5.87, and the fat-free formulation (F3), which scored 5.74 (Table 6).

A similar result was reported by Favaro-Trindade et al. [31] in a study on fermented acerola ice cream, where the aroma attribute received scores ranging between the categories “indifferent” and “slightly liked.” The authors suggested that the aroma could be improved by incorporating flavoring additives. In the present study, even with the use of 40% acerola pulp in the ice cream formulation, many panelists reported not perceiving the characteristic flavor of the fruit.

In contrast, da Paz et al. [59] evaluated the effect of adding dehydrated acerola (*Malpighia emarginata* DC) on the sensory characteristics of acerola ice cream and reported an overall acceptance score above 7 points. This study was conducted in Ceará, one of Brazil’s largest acerola-producing regions [60], which may have positively influenced product acceptance, particularly due to the addition of dehydrated acerola that enhances the fruit’s aromatic compounds.

Tolve et al. [61] demonstrated that incorporating fibers such as inulin and acacia into low-fat vanilla ice creams did not significantly affect their sensory characteristics compared to traditional formulations. This highlights the potential of using dietary fibers in the development of healthier, reduced-fat products without compromising sensory quality. However, unlike the present study, significant differences in sensory characteristics were observed.

Regarding purchase intent, all samples received average ratings corresponding to “maybe yes/maybe no”, although significant differences (*p* < 0.05) were found among the formulations. The samples containing fat (F1 and F2) did not differ from each other (*p* > 0.05), nor did the samples with PHGG (F2 and F3) (*p* > 0.05). This suggests that the intermediate formulation (F2) balanced desirable attributes from the high-fat formulation (F1) and the effects of fat replacement by PHGG in the fat-free formulation (F3), resulting in an intermediate purchase intent score (mean value of 3.5).

In general, the samples with higher fat content showed better sensory acceptance. Specifically, for appearance and texture attributes, the high-fat formulation (F1) received the highest acceptance scores among panelists, underscoring the role of fat in enhancing product perception. This preference may be associated with the lower overrun observed in the higher-fat formulation and the greater amount of cream, which positively influenced texture due to the creaminess and palatability provided by fat. Similarly, Hanafi et al. [62] reported lower acceptance for ice creams with higher dietary fiber content, attributed to negative impacts on texture and flavor.

## 4. Conclusions

Partial replacement of fat by PHGG proved to be a viable strategy for developing low-fat ice creams without compromising the physicochemical and microbiological quality of the product. The increase in total soluble solids was expected, considering the nature of PHGG as a soluble carbohydrate.

The composition of the ice creams changed as expected, with increased carbohydrate content and proportional reduction of lipids, while moisture and ash remained stable. The addition of PHGG influenced the rheological properties of the mix, leading to increased air incorporation (overrun), which is commonly associated with lighter texture characteristics. Despite these changes, fat replacement did not significantly affect the melting behavior of the ice cream, indicating that PHGG was able to maintain the structural stability of the system under the evaluated conditions.

The viability of *L. rhamnosus* GG was maintained above 8 log CFU/g throughout 180 days of storage, ensuring the probiotic potential of the product. The probiotic’s resistance to enteric phases I and II evaluated in vitro after 180 days of storage was limited, suggesting the need for strategies such as microencapsulation to optimize its survival in the gastrointestinal tract. Nonetheless, ice creams containing PHGG showed greater protection for the probiotic compared to the formulation without this fiber. The presence of the fiber itself already evidences the product’s prebiotic potential.

From a sensory perspective, the formulations showed satisfactory acceptance regarding aroma and flavor, with no differences among them. However, limitations in appearance and texture were identified, indicating the need for formulation adjustments to enhance sensory perception.

## Figures and Tables

**Table 1:** Formulations of acerola ice creams developed.

Ingredients	F1 (%)	F2 (%)	F3 (%)
Water	16.4%	20.4%	24.4%
Skim milk powder	9.0%	9.0%	9.0%
Sugar	14.0%	14.0%	14.0%
Milk cream (58% fat)	20.0%	10.0%	0.0%
Acerola pulp	40.0%	40.0%	40.0%
PHGG	0.0%	6.0%	12.0%
Stabilizer and emulsifier	0.5%	0.5%	0.5%
Probiotic	0.1%	0.1%	0.1%
Total	100%	100%	100%

F1 = formulation without PHGG (control) (0%); F2 = formulation with partial fat replacement and PHGG addition (6%); F3 = formulation with total fat replacement and PHGG addition (12%). PHGG: Partially hydrolyzed guar gum.

**Table 2:** pH, titratable acidity, and total soluble solids of acerola ice cream with partial and total fat replacement by PHGG and added *L. rhamnosus* GG.

Sample	pH		Acidity (% Lactic Acid)		Total Soluble Solids (°Brix)	
	1d	180d	1d	180d	1d	180d
F1	4.71 ± 0.16 ^Aa^	4.72 ± 0.13 ^Aa^	0.71 ± 0.06 ^Aa^	0.75 ± 0.05 ^Aa^	26.2 ± 1.6 ^Ca^	27.1 ± 1.1 ^Ca^
F2	4.66 ± 0.15 ^Aa^	4.73 ± 0.06 ^Aa^	0.73 ± 0.06 ^Aa^	0.70 ± 0.04 ^Aa^	30.8 ± 1.3 ^Ba^	30.0 ± 1.6 ^Ba^
F3	4.65 ± 0.09 ^Aa^	4.67 ± 0.09 ^Aa^	0.73 ± 0.10 ^Aa^	0.73 ± 0.04 ^Aa^	34.2 ± 1.6 ^Aa^	34.0 ± 1.5 ^Aa^

Different uppercase letters indicate significant differences between formulations (columns) at the same time point according to Tukey’s test at 5% probability (*p* < 0.05); different lowercase letters indicate significant differences between time points (rows) for the same formulation according to the same statistical test. PHGG: Partially hydrolyzed guar gum. F1: 0% PHGG; F2: 6% PHGG; F3: 12% PHGG; d: day(s).

**Table 3:** Proximate composition of acerola ice cream with partial and total fat replacement by PHGG and added *L. rhamnosus* GG.

Sample	Moisture (%)		Carbohydrates * (%)		Lipids (%)		Proteins (%)		Ash (%)	
	1d	180d	1d	180d	1d	180d	1d	180d	1d	180d
F1	63.4 ± 1.3 ^Aa^	62.5 ± 1.2 ^Aa^	20.5 ± 1.4 ^Ca^	22.0 ± 1.7 ^Ca^	11.5 ± 1.1 ^Aa^	11.2 ± 1.3 ^Aa^	4.1 ± 0.2 ^Aa^	3.3 ± 0.5 ^Ab^	0.94 ± 0.06 ^Aa^	1.01 ± 0.12 ^Aa^
F2	64.0 ± 1.2 ^Aa^	62.9 ± 1.1 ^Aa^	25.0 ± 1.3 ^Ba^	27.2 ± 2.1 ^Ba^	5.9 ± 0.8 ^Ba^	6.1 ± 1.3 ^Ba^	4.0 ± 0.3 ^Aa^	3.0 ± 0.3 ^Ab^	0.93 ± 0.04 ^Aa^	0.90 ± 0.04 ^Aa^
F3	63.2 ± 1.6 ^Aa^	63.4 ± 1.4 ^Aa^	31.6 ± 0.4 ^Aa^	32.3 ± 1.2 ^Aa^	0.2 ± 0.1 ^Ca^	0.3 ± 0.2 ^Ca^	3.9 ± 0.2 ^Aa^	3.1 ± 0.4 ^Ab^	0.96 ± 0.02 ^Aa^	0.94 ± 0.02 ^Aa^

* Total carbohydrate content obtained by difference, including dietary fiber (PHGG). Different uppercase letters indicate significant differences between samples (column) at the same time according to Tukey’s test at 5% probability (*p* < 0.05), and lowercase letters indicate significant differences between storage times (row) for the same sample according to the same statistical test. PHGG: Partially hydrolyzed guar gum. F1: 0% PHGG; F2: 6% PHGG; F3: 12% PHGG; d: day(s).

**Table 4:** Overrun, melting properties, and density of acerola ice cream with partial and total fat replacement by PHGG and added *L. rhamnosus* GG after 1 day of production.

Sample	Overrun (%)	Melting Rate (g/min)	Time to First Drop (min)	Density (g/L)
F1	27.8 ± 3.7 ^C^	1.93 ± 0.10 ^A^	10.0 ± 3.2 ^A^	919 ± 5.2 ^A^
F2	42.0 ± 3.3 ^B^	1.82 ± 0.21 ^A^	9.7 ± 3.8 ^A^	811 ± 5.6 ^B^
F3	89.4 ± 13.0 ^A^	2.06 ± 0.11 ^A^	8.2 ± 2.1 ^A^	631 ± 46.7 ^C^

Different uppercase letters indicate significant differences among samples in the same column according to Tukey’s test at 5% probability (*p* < 0.05). PHGG: Partially hydrolyzed guar gum. F1: 0% PHGG; F2: 6% PHGG; F3: 12% PHGG.

**Table 5:** Rheological properties of acerola ice cream mixes with partial and total fat replacement by PHGG and addition of *L. rhamnosus* GG evaluated at 7 °C.

Sample	Ostwald–de Waele Model			Apparent Viscosity (Pa s)		
	k (Pa·s^n^)	*n*	R^2^	γ = 10 (s^−1^)	γ = 50 (s^−1^)	γ = 100 (s^−1^)
F1	51.3 ± 3.3 ^b^	0.64 ± 0.01 ^b^	0.9992	22.4 ± 0.9 ^c^	12.6 ± 0.3 ^c^	9.8 ± 0.2 ^c^
F2	72.2 ± 4.7 ^a^	0.66 ± 0.01 ^b^	0.9989	33.3 ± 1.3 ^b^	19.4 ± 0.4 ^b^	15.3 ± 0.2 ^b^
F3	73.8 ± 6.4 ^a^	0.77 ± 0.01 ^a^	0.9997	43.1 ± 1.4 ^a^	29.6 ± 0.7 ^a^	25.2 ± 0.5 ^a^

Different lowercase letters in the same column indicate significant differences among treatments by Tukey’s test at 5% probability (*p* < 0.05). γ: Shear rate. PHGG: Partially hydrolyzed guar gum. F1: 0% PHGG; F2: 6% PHGG; F3: 12% PHGG.

**Table 6:** Sensory evaluation of acerola ice cream with partial and total fat replacement by PHGG and addition of *L*. *rhamnosus* GG.

Sample	Appearance	Aroma	Flavor	Texture	Overall Impression	Purchase Intention
F1	7.87 ± 2.00 ^A^	6.41 ± 2.01 ^A^	6.13 ± 2.35 ^A^	7.14 ± 2.37 ^A^	6.53 ± 2.19 ^A^	3.79 ± 1.25 ^A^
F2	7.14 ± 2.14 ^B^	6.03 ± 2.13 ^A^	5.45 ± 2.30 ^A^	6.29 ± 2.42 ^B^	5.87 ± 2.08 ^AB^	3.50 ± 1.22 ^AB^
F3	6.22 ± 2.29 ^C^	6.14 ± 2.01 ^A^	5.55 ± 2.20 ^A^	5.61 ± 2.31 ^B^	5.74 ± 2.03 ^B^	3.32 ± 1.12 ^B^

Different uppercase letters in the same column indicate significant differences among samples according to Tukey’s test at a 5% significance level (*p* < 0.05). PHGG: Partially hydrolyzed guar gum. F1: 0% PHGG; F2: 6% PHGG; F3: 12% PHGG.

## Data Availability

The data presented in this study are included in the article/Supplementary Materials; further inquiries can be directed to the corresponding author.

## Supplementary Materials

The following supporting information can be downloaded at: https://www.mdpi.com/article/10.3390/foods15122186/s1, Table S1. Mean values of microbiological counts (CFU/g) for ice cream formulations (F1, F2, F3) across three repetitions.
