# Supplementary material for: Production and Characterization of Potentially Symbiotic Acerola Ice Cream with Partially Hydrolyzed Guar Gum and Added Lacticaseibacillus rhamnosus GG
Source: Foods. 2026 Jun 17;15(12):2186. doi: 10.3390/foods15122186 (PMC13298192; doi:10.3390/foods15122186)
Supplement: Supplementary file 1 [file foods-15-02186-s001.zip › foods-4288893-supplementary.pdf]

Supplementary Material - Microbiological Analysis

Table S1. Mean values of microbiological counts (CFU/g) for ice cream formulations (F1, F2, F3) across three repetitions.

Formulation	Salmonella spp.	Enterobacteriaceae (CFU/g)	Coagulase-positive staphylococci (CFU/g)
F1	Absent	1.8×10^1	$< 1.0 \times 10^1$
F2	Absent	2.2×10^1	$< 1.0 \times 10^1$
F3	Absent	2.9×10^1	$< 1.0 \times 10^1$

Note: Values represent the mean of three repetitions.
